# Strengthening of Bent LVL Beams with Near-Surface Mounted (NSM) FRP Reinforcement

**DOI:** 10.3390/ma13102350

**Published:** 2020-05-20

**Authors:** Michał Marcin Bakalarz, Paweł Grzegorz Kossakowski, Paweł Tworzewski

**Affiliations:** Department of the Strength of Materials, Concrete Structures and Bridges, Faculty of Civil Engineering and Architecture, Kielce University of Technology, aleja Tysiąclecia Państwa Polskiego 7, 25-314 Kielce, Poland; kossak@tu.kielce.pl (P.G.K.); ptworzewski@tu.kielce.pl (P.T.)

**Keywords:** 4-point bending, veneer, wood structures, carbon fibers, reinforcement

## Abstract

The topic of the article is the analysis of the static work of unreinforced and reinforced with composite material timber beams under bending tests. The results of the experimental tests and a brief outline of the characteristics of the internal reinforcement of wood structures are presented. Experimental tests were performed on full-scale beams made of laminated veneer lumber (LVL) with nominal dimensions of 45 × 200 × 3400 mm. Two strips of carbon fiber-reinforced polymer (CFRP) reinforcement were glued into rectangular grooves in the component bottom with two-component epoxy resin (0.62% reinforcement percentage). The reinforcement mainly affected the enhancement of the maximum bending moment values evaluated at the points of application as having concentrated forces of 32% and 24% in comparison to the unreinforced elements. Increases of 11% and 7% in the global modulus of elasticity in the bending and stiffness coefficients were achieved, respectively. The failure of the reference beams was caused by exceeding the tensile strength of the LVL. The reinforced elements were characterized by a greater variation in failure mode, resulting from tension, compression or lateral torsional buckling. The strain profile reading showed a higher utilization of the compression characteristic of veneer in specimens reinforced with carbon laminates.

## 1. Introduction

Reinforcing timber beams with glued-in fiber-reinforced polymer inside the cross-section provides an aesthetic external surface for the reinforced beam, exposing the structure of the timber and protecting the reinforcement against environmental factors. Due to the position of the reinforcement in relation to the original cross-section of the reinforced beam, the near-surface mounted and internal reinforcement should be distinguished. Near-surface mounted (NSM) fiber-reinforced polymer (FRP) reinforcement is done by hollowing out a groove, or grooves, within the cross-section perimeter in which the reinforcement inserts are then placed and an adhesive composition poured on. In this way, the outer layer of the adhesive is flush with the outer surface of the reinforced component [1,2]. In the second case, the reinforcement is completely surrounded—this can be achieved by moving the reinforcement deep into the cross-section or by using additional wooden cover plates glued to the applied reinforcement. 

Previous attempts to apply internal reinforcement to wood structures included the introduction of reinforcement inserts into the tensile zone or into the tensile and compression zone—a more effective method [3,4]. Both metallic, as well as fiber-reinforced polymer, reinforcement in the form of rods [5,6,7,8], laminates or mats were used [9,10,11]. The use of carbon fiber-reinforced polymer (CFRP) laminates bonded inside the cross-section perimeter has been presented [12] as an implementable solution for strengthening and reproducing the carrying capacity of solid timber beams in historical structures. The strengthening of glued laminated timber (glulam) elements at the component production stage with one layer of glass-aramid tape bonded internally has been described in [13] as beneficial where the strength of material decides about load carrying capacity. The reinforcement of low-grade, glued laminated timber that has been bonded in circular grooves at the bottom of beam basalt rods has been investigated experimentally [14]. A mean stiffness enhancement of 8.4% and 10.3% for the global and local stiffnesses, respectively, and a 23% increase in the ultimate bending moment have been achieved with a modest reinforcement percentage of 1.4%. Passive and active reinforcement of glulam beams using carbon fiber-reinforced polymer bars bonded in rectangular grooves has been presented [15]. Great improvements in flexural capacity and bending stiffness were obtained.

In addition to the scale and location of reinforcement, the strengthening performance was significantly affected by the mechanical properties of the reinforced component. The higher increase in flexural strength is achieved for the beams with lower mechanical properties.

Limited research has been undertaken into the use of composite materials to strengthen laminated veneer lumber (LVL) beams. The objective of this study is to determine the effectiveness of strengthening the LVL beams with the use of CFRP strips bonded in rectangular pre-drilled grooves in the tension zone. The scope of the test program included fabrication and performing 4-point bending tests on unreinforced and reinforced specimens. The static work of the unreinforced and reinforced specimens under bending is compared with regard to the load–deflection and load–time behavior, failure mode, enhancements in global modulus of elasticity in bending, stiffness coefficient, maximum bending moments as evaluated at the points of application of concentrated forces and strain distribution. The test results are a continuation of the work on reinforcing LVL beams with fiber-reinforced polymer materials. In other works [16,17], issues of reinforcing laboratory scale specimens by gluing aramid, carbon and glass sheets to the external surfaces of the components were raised. 

## 2. Materials and Methods 

The reinforced beams were laminated veneer lumber beams with a unidirectional fiber system. The beams were examined in the edgewise condition. The nominal dimensions of the beams were 45 × 200 × 3400 mm. Grooves that were 25 mm deep, 12 mm wide and 2850 mm long were cut out along the bottom surface. The groove edges were rounded. The grooves were located symmetrically to the vertical axis of the cross-section and along the beam. The basic beam parameters are shown in Table 1.

Rectangular laminates reinforced with 20 mm wide and 1.4 mm thick carbon fibers, cut to 2800 mm in length, were applied as reinforcement inserts. Two reinforcement inserts were placed in each groove—vertically next to each other. The spacing of the inserts along the hole was adjusted with steel wire spacers. In this way, a 0.62% reinforcement percentage was obtained. The basic reinforcement parameters are shown in Table 2.

Two-component epoxy resin was used to glue the inserts into the cross-section. Selected parameters of epoxy resin are shown in Table 3. The beams remained in an inverted system during the hole pouring and drying process. 

**Figure 1:**
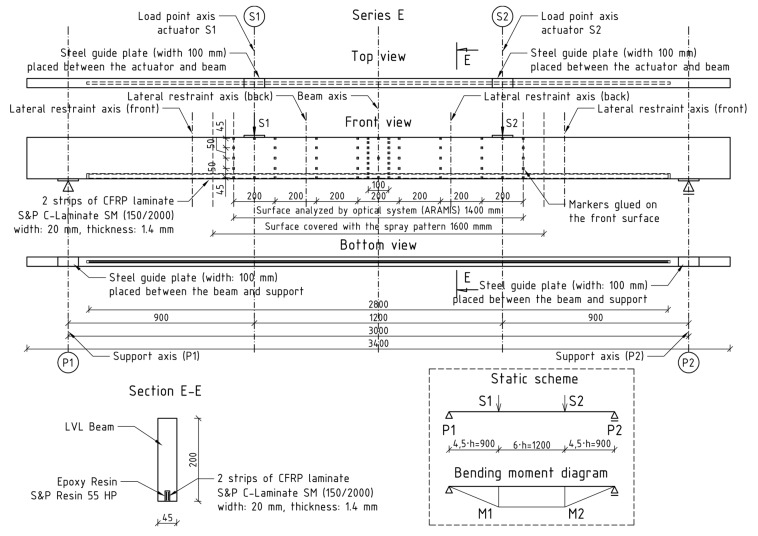
E-series beam reinforcement diagram with marked support and protection axes.

**Figure 2:**
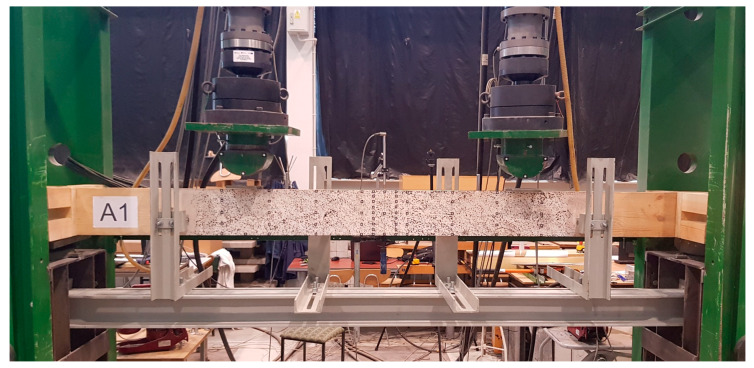
View of the static test setup.

The tests were performed in the Materials Strength Laboratory at the Kielce University of Technology in accordance with the guidelines of the standards [18,19]. The tests covered the so-called 4-point bending to destroy two series of components: series A, test beams, and series E, beams reinforced with two CFRP laminate strips glued into the grooves from below. Both series included five components. The static diagram and view of the test bench are shown in Figure 1 and Figure 2.

Steel guide plates of 10 mm thickness, 100 mm width and 200 mm length were placed at supports and under the loading heads to prevent local indentation. Four lateral restraints, in form of roller support, were used to prevent lateral torsional buckling—two on each side of the specimen. The lateral restraints were localized approximately 300 mm from the load point axis (Figure 2). The lateral restraint spacing was adjusted to the width of the loading head and the surface covered with the spray pattern. Actuators (S1 and S2) were controlled individually by a displacement rate of 7 mm/min. This resulted in differences between the recorded values of forces between the actuators and thus changed the standard shape of the cross-sectional force diagrams—bending moments and shearing forces. 

The tests recorded the loading force value for each actuator, displacement of the actuators, test time and beam deflection in the beam span center in the upper fibers compressed using an inductive sensor. On the basis of these parameters, the maximum bending moment and global modulus of elasticity at bending were determined and the work of the tested components analyzed. After the tests, the moisture content of the components was verified with a resistance-type hygrometer, and the component failure method was documented. 

Additionally, the displacements and deformations of the middle part of the specimens were recorded with the use of an optical measurement system (ARAMIS). The front surface of the beam, which was 1600 mm wide, was covered with a spray pattern. Markers glued on the front surface (Figure 1) acted as reference points.

The global modulus of elasticity at bending was determined by the following equation [18]:

(1) $$E_{m,g}=\frac{3al^{2}-4a^{3}}{2bh^{3}\left(2\frac{w_{2}-w_{1}}{F_{2}-F_{1}}-\frac{6a}{5Gbh}\right)}$$

where:

*F*_2_ − *F*_1_: applied load increment within the linear regime (N);

*w*_2_ − *w*_1_: deflection increment corresponding to the load increment (mm);

*G*: shear modulus (assumed according to the manufacturer data).

The stiffness coefficient of the beam was determined within the elastic range according to the equation:

(2) $$k=\frac{F}{u}$$

where:

*F*: loading force (kN);

*u*: deflection corresponding to the loading force (mm).

## 3. Results

The general outline of the tests is presented by means of the total load relationship diagrams understood as the sum of the forces recorded by the computer set in each of the actuators with respect to the test duration and the deflection at the center of the beam span. The maximum force values, the determined parameters and the basic statistical analysis are summarized in Table 4 and Table 5.

**Figure 3:**
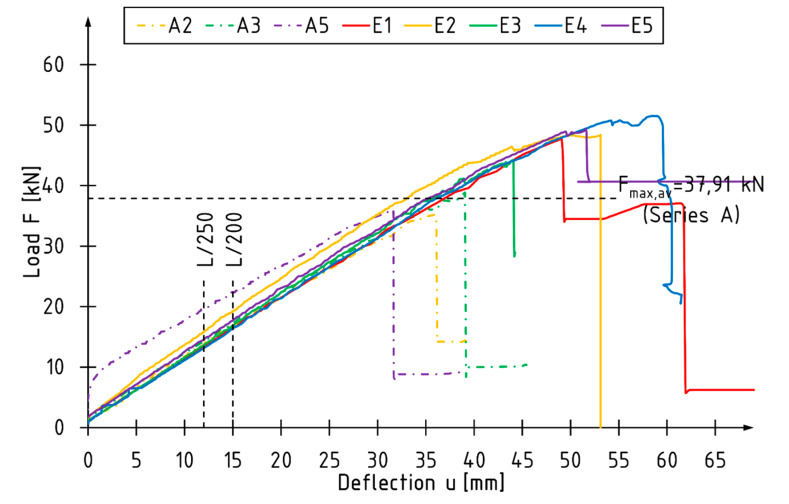
The total load–deflection diagram for selected beams.

Near-surface mounted (NSM) FRP reinforcement changed the behavior of components during bending. In the case of the A-series, the shape of individual curves is almost rectilinear. An increase in the ductility can be observed for the E-series components—curvature of the curves in the final phase of the tests (Figure 3). 

**Figure 4:**
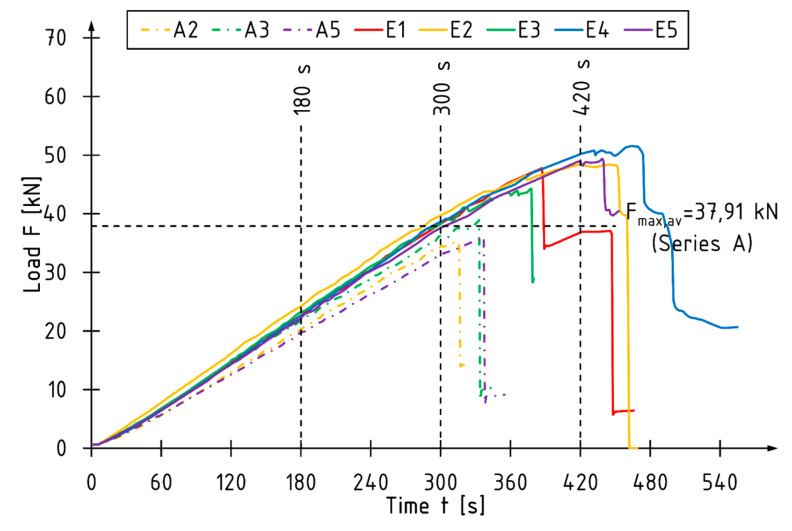
The total load–time diagram for selected beams.

For all the reference beams, the time taken to achieve the maximum total load is reached within the time interval recommended by the standard [16]. The beams E2, E4 and E5 were destroyed in a time slightly surpassing this area (Figure 4).

**Figure 5:**
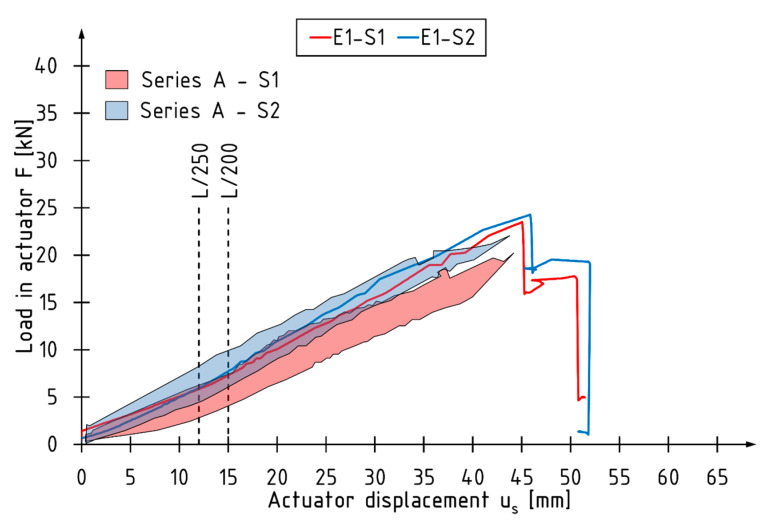
Change in force values recorded during the test for the E1 beam.

Figure 5 shows a typical course of changes in force values in individual actuators as a function of displacement for the E1 beam. The area of recorded force values in the actuators for the A-series beams is plotted in the background. For unreinforced beams, higher force value was recorded in the actuator S2. 

**Figure 6:**
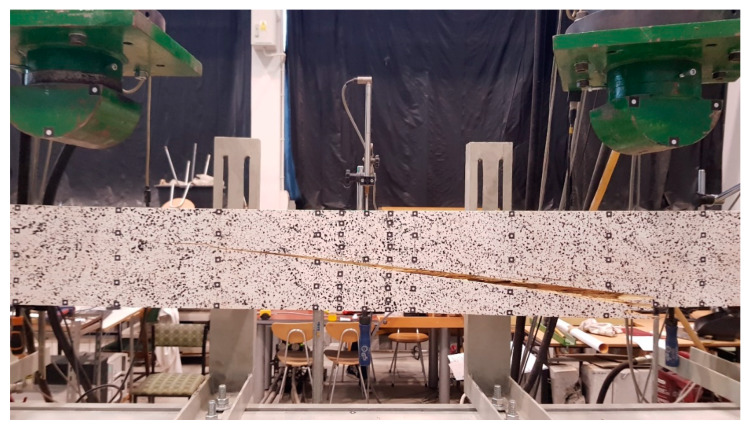
Typical failure mode of a reference beam (tension).

The failure of the reference components was due to the exhaustion of the tensile strength in the extreme tension fibers. The failure initiation followed in the maximum bending moment zone between the points of application of the load thrusts. An example of a typical failure of a reference beam is shown in Figure 6. 

**Figure 7:**
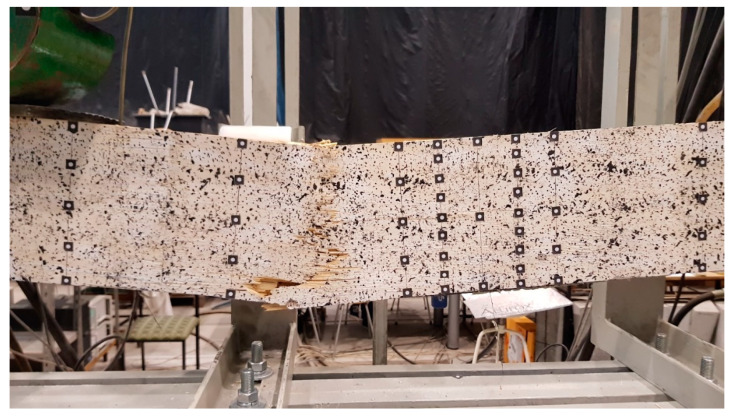
An example of a reinforced beam failure (tension + compression).

The reinforced beams were characterised by a much greater variation in the form of failure. This was mainly due to the resistance exhaustion in the veneer. The failure initiation occurred in the tensile or compression zone (Figure 7). In the case of the E2 component, the failure of the veneer was accompanied by the breaking out of the carbon laminates. The CFRP laminate did not break in any of the tested components.

The table below shows detailed test results for the tested beams. 

The average maximum loading force of reinforced beams is 27% higher than that of reference beams. The average increases in the global modulus of elasticity in the bending and stiffness coefficients are 11% and 7%, respectively. 

**Figure 8:**
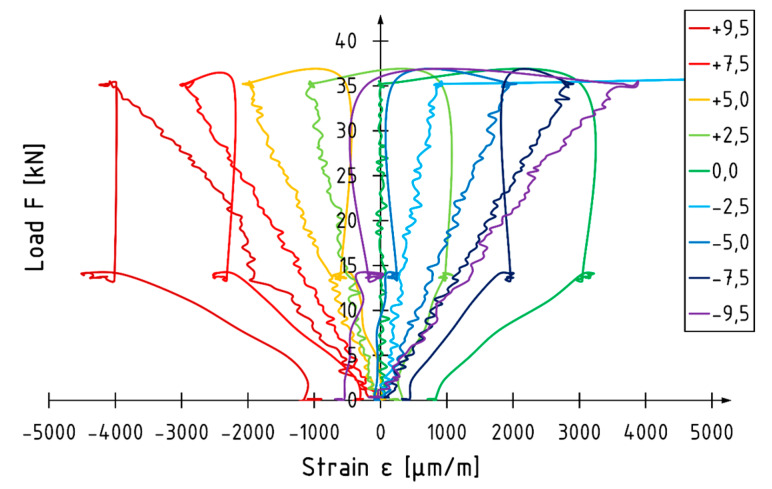
Load–strain diagram for beam A1.

**Figure 9:**
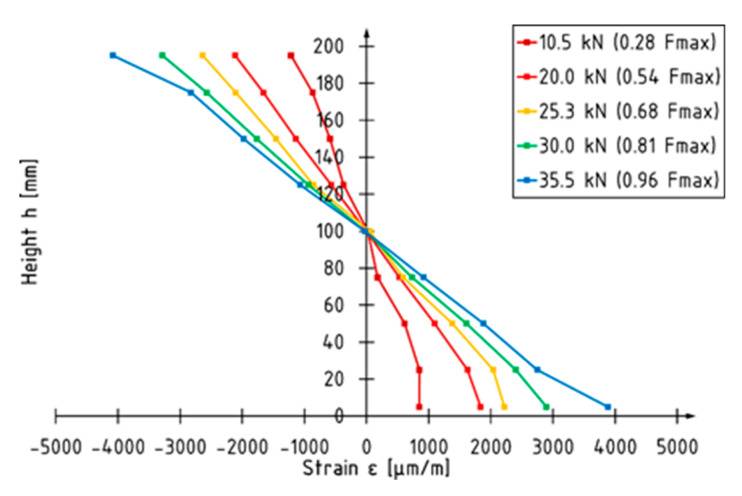
Strain profile for beam A1.

A typical load versus strain diagram for unreinforced specimens is shown in Figure 8 (beam A1). Curves present strain values measured at the front surface in the middle of specimen. Strain measurements were performed using an optical measurement system (ARAMIS, GOM GmbH, Braunschweig, Germany) until the loading force was removed. Each curve corresponds to the level on which markers were glued (Figure 1). The depth of the level is measured from the centroid of the beam. Unreinforced elements are characterized by linear strain behavior. The neutral axis is localized at the centroid of the beam regardless of the value of the loading force. The strain profile for beam A1 is shown in Figure 9. The maximum strain recorded at the applied force of 35.5 kN (corresponding to 96% of maximum loading force recorded during the test) was −4086 and 3884 for the compression and tension zone, respectively.

**Figure 10:**
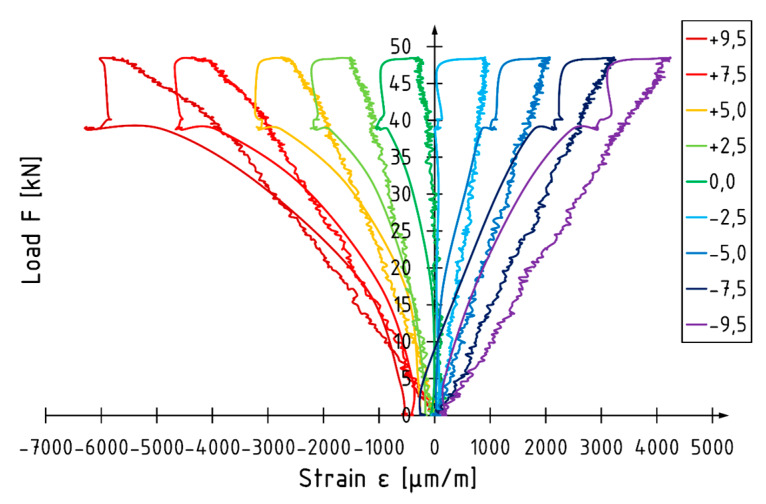
Load–strain diagram for beam E5.

**Figure 11:**
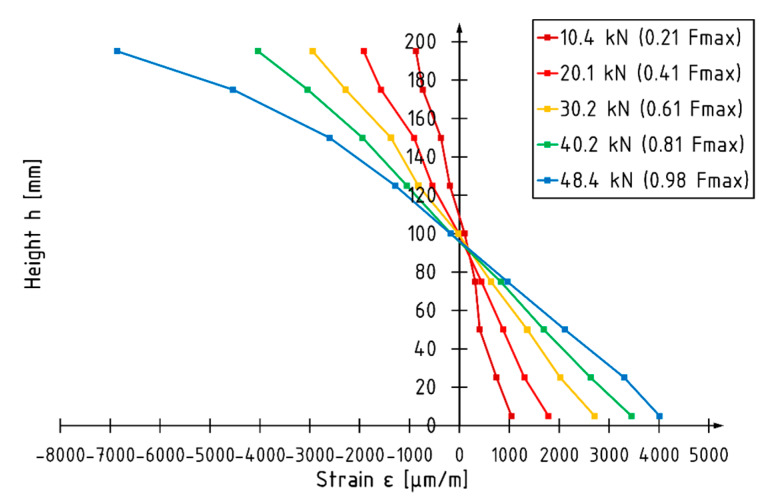
Strain profile for beam E5.

The load versus strain diagram for beam E5 is shown in Figure 10. Non-linear strain behavior is recorded in the final phase of the test in the compression zone. Strain behavior in the tension zone is linear up to failure. The position of the neutral axis changes depending on the value of the loading force. When there is an applied load of approximately 30 kN, the neural axis deepens in the cross-section. The strain profile for beam E5 is shown in Figure 11. When there is an applied force of 48.4 kN, corresponding to 98% of the maximum loading force recorded, the neutral axis is approximately 6% lower compared to the depth of the centroid of the beam. The maximum strain recorded at the load level was −6859 and 4012 for the compression and tension zone respectively. A higher utilization of the compression characteristics of veneer can be distinguished for reinforced elements.

Results of the analytical analysis for the E5 beam are shown in Table 6. The transformed cross-section method was used to evaluate the bending stiffness and stress in the LVL at the centroid of the reinforcement inserts at selected levels of loading force. The average value of the bending moment was used to determine the stress in the LVL. The stress in the CFRP laminate was determined assuming that the strain in the LVL and reinforcement was equal. 

For reinforced elements, the neutral axis is approximately 7% lower when compared to the depth of the centroid of the beam for the transformed cross-section. The average value of the stress in CFRP laminates was 610 MPa, which corresponds to 22% of its tensile strength according to the data provided by manufacturer. Stress values determined on the basis of the test results are higher, approximately 6% in the final phase of the test, when compared to the values determined according to the transformed cross-section method.

## 4. Discussion and Conclusions

The article presents the results of experimental tests on reinforcing narrow beams with carbon fiber-reinforced laminates placed into grooves at the bottom of the beam. Two laminate strips were placed in each groove. In conclusion:

1. The control of the load speed of the actuators by means of a fixed displacement rate may cause disturbances in the cross-sectional force values compared to the standard 4-point bending test distribution. A simultaneous loading of the beam with two separately controlled concentrated forces allows for more accurate descriptions of the work of the beams before and immediately after their failure in terms of the working load and its values for individual zones.
2. For the unreinforced beams, the maximum loading force and thus the bending moment were usually reached at the actuator number S2, so the average value of the bending moment *M*2 is 6% higher than the bending moment *M*1.
3. The reinforcement with CFRP laminates glued into the grooves mainly resulted in an increase in the maximum bending moment of the beams—32% and 24% compared to the reference beams for *M*1 and *M*2 values, respectively.
4. The use of a 0.62% reinforcement ratio resulted in enhancing the global modulus of elasticity in the bending and stiffness coefficients by 11% and 7%, respectively.
5. The reinforced beams were characterized by more ductile behavior under bending (non-linear behavior for E2, E4, and E5 beam observed on load versus deflection diagram in the final phase of the tests), which can be equated with greater use of the compression zone of the cross-section.
6. Unreinforced elements failure was caused by exceeding the tensile strength. Various forms of beam failure were observed for the reinforced beams, with the initiation of most of them resulting from exceeding the veneer strength in the compression or tensile zone. The CFRP laminates were not destroyed.
7. The strain profile reading showed higher utilization of compression characteristics of the LVL in reinforced elements.
8. The CFRP laminates used as a reinforcement in the current study are relatively expensive in comparison to conventional materials (steel plates, rods) or composites reinforced with glass or aramid fiber. However, they are characterized by great fatigue behavior and corrosion resistance. The CFRP laminates are considerably lighter and have better mechanical parameters when compared to steel.
9. The proposed strengthening method is suitable for enhancing the mechanical parameters of existing elements.

## Figures and Tables

**Table 1:** Selected physical and mechanical parameters of laminated veneer lumber (LVL) beams (exposed by manufacturer).

Parameter	Edgewise Condition
Bending strength (N/mm^2^)	44
Tensile strength (N/mm^2^)	36
Compression strength parallel to the grains (N/mm^2^)	40
Shear strength (N/mm^2^)	4.6
Young’s Modulus (N/mm^2^)	14,000
Shear Modulus (N/mm^2^)	600
Density (kg/m^3^)	480

**Table 2:** Physical and mechanical parameters of carbon laminates (exposed by manufacturer).

Parameter	CFRP Laminates
Width/Thickness/Length (mm)	20/1.4/2800
Modulus of elasticity (kN/mm^2^)	170
Tensile strength (N/mm^2^)	2800
Density (g/cm^3^)	1.6
Fiber volume (%)	68
Elongation at rupture (‰)	16

**Table 3:** Selected physical and mechanical parameters of epoxy resin (exposed by manufacturer).

Parameter	Epoxy Resin
Modulus of elasticity in compression (N/mm^2^)	3200
Compressive strength (N/mm^2^)	100
Density (g/cm^3^)	1–1.1
Elongation at break (‰)	17.3

**Table 4:** Test results.

No.	Loading Force (kN)	Max. Bending Moment (kNm)		Stiffness Coefficient (kN/mm)	MOE (GPa)	Global Stiffness EI (Nmm^2^)	Failure Mode
		*M*1	*M*2				
A1	36.86	15.86	17.32	0.89	12.73	3.98 × 10^8^	Tension
A2	35.33	15.33	16.46	1.14	14.50	4.52 × 10^8^	Tension
A3	38.89	17.30	17.70	1.05	14.68	4.49 × 10^8^	Tension
A4	42.36	18.71	19.41	1.04	15.09	4.60 × 10^8^	Tension
A5	36.08	15.54	16.93	0.90	13.77	3.99 × 10^8^	Tension
E1	47.85	21.39	21.67	1.02	14.84	4.55 × 10^8^	Tension
E2	48.44	21.23	22.60	1.15	16.42	5.11 × 10^8^	Compression + Tension
E3	44.36	20.78	19.22	1.07	15.76	4.76 × 10^8^	LTB ^1^
E4	51.54	23.70	23.41	1.04	15.62	4.63 × 10^8^	Compression
E5	49.41	22.66	22.12	1.07	15.70	4.76 × 10^8^	Compression

^1^ Lateral torsional buckling.

**Table 5:** Averages values of selected, recorded and evaluated parameters.

Series	Loading Force (kN)	Max. Bending Moment (kNm)		Time to Failure (s)	Stiffness Coefficient (kN/mm)	Modulus of Elasticity (GPa)	Global Stiffness EI (Nmm^2^)
		*M*1	*M*2				
A	37.91	16.55	17.56	338.1	1.00	14.16	4.26 × 10^8^
E	48.32 (+27%)	21.78 (+32%)	21.71 (+24%)	422.6 (+25%)	1.07 (+7%)	15.67 (+11%)	4.96 × 10^8^ (+16%) (4.76 × 10^8^) ^1^

^1^ Average value of bending stiffness determined on the basis of the transformed cross-section.

**Table 6:** Analytical analysis for beam E5.

Loading Force (kN)	Stress in LVL *σ*1 (MPa) ^1^	Stress in CFRP Laminate (MPa) ^1^	Stress in LVL *σ*2 (MPa) ^2^	Stress Ratio *σ*1/*σ*2
10.2	13.03	158.16	10.67	1.22
20.2	22.44	272.53	20.65	1.09
30.2	34.32	416.74	31.02	1.11
40.1	43.99	534.17	41.32	1.06
46.6	52.97	643.16	49.73	1.06

^1^ Values determined on the basis of the test results. ^2^ Values determined on the basis of the transformed cross-section.
